# Examining the Use of Autonomous Systems for Home Health Support Using a Smart Mirror

**DOI:** 10.3390/healthcare11192608

**Published:** 2023-09-22

**Authors:** Liz Dowthwaite, Gisela Reyes Cruz, Ana Rita Pena, Cecily Pepper, Nils Jäger, Pepita Barnard, Ann-Marie Hughes, Roshan das Nair, David Crepaz-Keay, Sue Cobb, Alexandra Lang, Steve Benford

**Affiliations:** 1 Horizon Digital Economy Research, University of Nottingham, Nottingham NG7 2TU, UKpepita.barnard@nottingham.ac.uk (P.B.); 2 School of Computer Science, University of Nottingham, Nottingham NG8 1BB, UK; steve.benford@nottingham.ac.uk; 3 Horizon Centre for Doctoral Training, University of Nottingham, Nottingham NG8 1BB, UK; ppyarpe@nottingham.ac.uk (A.R.P.); psxcp3@nottingham.ac.uk (C.P.); 4 Department of Architecture and Built Environment, University of Nottingham, Nottingham NG7 2RD, UK; nils.jaeger@nottingham.ac.uk; 5 School of Health Sciences, University of Southampton, Southampton SO17 1BJ, UK; a.hughes@soton.ac.uk; 6 Faculty of Medicine and Health Sciences, University of Nottingham, Nottingham NG7 2UH, UK; roshan.nair@sintef.no; 7 Health Division, Stiftelsen for Industriell og Teknisk Forskning (SINTEF), 0314 Oslo, Norway; 8 Mental Health Foundation, London SE1 4PD, UK; dcrepaz-keay@mentalhealth.org.uk; 9 Faculty of Engineering, University of Nottingham, Nottingham NG7 2RD, UK; susan.cobb@nottingham.ac.uk (S.C.);

**Keywords:** autonomous systems, multiple sclerosis, stroke, smart mirror, autonomy, lived experience, home healthcare, digital health technology, data sharing

## Abstract

The home is becoming a key location for healthcare delivery, including the use of technology driven by autonomous systems (AS) to monitor and support healthcare plans. Using the example of a smart mirror, this paper describes the outcomes of focus groups with people with multiple sclerosis (MS; *n* = 6) and people who have had a stroke (*n* = 15) to understand their attitudes towards the use of AS for healthcare in the home. Qualitative data were analysed using a thematic analysis. The results indicate that the use of such technology depends on the level of adaptability and responsiveness to users’ specific circumstances, including their relationships with the healthcare system. A smart mirror would need to support manual entry, responsive goal setting, the effective aggregation of data sources and integration with other technology, have a range of input methods, be supportive rather than prescriptive in messaging, and give the user full control of their data. The barriers to its adoption include a perceived lack of portability and practicality, a lack of accessibility and inclusivity, a sense of redundancy, feeling overwhelmed by multiple technological devices, and a lack of trust in data sharing. These results inform the development and deployment of future health technologies based on the lived experiences of people with health conditions who require ongoing care.

## 1. Introduction

Exacerbated by the COVID-19 pandemic, many activities that were previously undertaken elsewhere now take place within the home, including work, education, and healthcare. Home healthcare technologies have become increasingly popular, such as devices for monitoring blood pressure, heart rate, and diabetes, smart toothbrushes for supporting oral health, and a range of mobile apps and wearables to aid general fitness. The collection and provision of data pertaining to personal health and wellbeing with the absence of professional clinical support could be considered a limitation of these products. However, these technologies give people ownership of information about their own activities, providing them with the opportunity to increase their awareness of their lifestyle patterns, and even suggest changes to improve them. They are often used to facilitate and make autonomous decisions regarding healthcare delivery for both healthy populations and people with a variety of health conditions. Despite the potential for concerns around privacy and security associated with these technologies, they are starting to contribute tangibly to the healthcare sector by offering novel data sets for use in user–clinician interactions and consultations. In addition, many general practitioners (GPs) and other healthcare professionals now offer routine appointments via video conferences. These technology-enabled and -enhanced approaches to healthcare delivery offer new opportunities for research into the experience and design of healthcare experiences at home.

Notably, healthcare technologies used at home are usually portable or wearable, whereas devices embedded in the home space remain underexplored and are not yet widely adopted. This paper describes the results of a study exploring how such embedded autonomous systems (ASs) can support decision-making about health and wellbeing, and what makes these devices acceptable to users. By engaging with people who have had a stroke and those who have multiple sclerosis (MS), this study examined attitudes towards the use of ASs in decision-making related to their health and wellbeing, especially how this relates to shared values such as trust, self-efficacy, and privacy. These two populations were chosen because they experience distinct but similar health-management challenges in their everyday lives and need to manage their multiple symptoms daily. People in both groups often experience the following, to a greater or lesser extent: memory problems, gait and mobility challenges, mental health issues, fatigue, communication problems, and muscle and neuropathic pain.

To explore how ASs could and should be designed to support healthcare at home, and to make engagement with this topic more tangible, online focus groups using the idea of a ‘smart mirror’ as a technology probe were conducted. This mirror was envisioned as a piece of technology installed in a user’s home, in areas such as the bathroom, bedroom, or entrance hall, which would be able to visually assess a person with a medical condition and carry out a range of tasks to support them in the management of their symptoms, including the following: monitoring their physical and psychological state, suggesting modifications to their treatment plan, interfacing with carers (medical professionals, family, close friends), and helping with daily activities, such as providing reminders for appointments, activities, and medication. The focus groups revealed a range of important considerations for the deployment of AS in the home, within the broader contexts of digital healthcare and self-management and of the home as a space for wellbeing.

### 1.1. Digital Healthcare

Both inside and outside the home, people make wide use of technology, such as smartphones and wearable technology, for the self-tracking of their health and to enhance their self-knowledge, in what has been termed the ‘quantified self’ [1,2,3]. Numerous aspects of health can be tracked using smart technology, including activity levels, heart rate, hours of sleep, and even stress levels; many people use these to maintain their general health or to assist them in reaching fitness or health-related goals. The technology sector encourages this idea of the quantified self, with all smartphones now equipped with GPS (global positioning system) technology and health-related apps to facilitate self-tracking [4]. While this has multiple benefits in terms of the promotion of health, novel smart technology presents challenges in the areas of security, privacy, and trust [5]. Thus, research exploring these facets of novel technology that aims to improve digital healthcare is crucial.

Autonomous systems hold great promise for improving healthcare, including patient outcomes, cost reductions and enabling new medical discoveries [6]. These technologies have the potential to analyse tasks beyond human capability [7], as well as the potential to reduce the high level of health and social care needs associated with increasing populations with complex and long-term conditions and with ageing populations [8]. Various health conditions can be assessed and tracked by wearable technology and apps, including type 2 diabetes, hypertension, fertility, concussion detection, traumatic brain injury, skin cancer, and mental health issues [9,10,11,12,13,14].

This paper describes a study exploring two populations: people who have MS and people who have had a stroke. Digital healthcare technology (primarily sensor-based equipment or wearable technology) is used to monitor specific MS symptoms such as poor mobility and balance, which contribute to the increased likelihood of falls and decreased quality of life [15], conveying promising results [16]. Other research has shown that individuals with MS may benefit from such technologies due to the feeling of regaining control over unpredictable conditions through self-tracking and improved self-knowledge [17]. Furthermore, technologies have developed in post-stroke care, including the detection of gait and the analysis of movement, assessments and guiding exercises to help with balance complications, and the tracking of upper-limb rehabilitation [18,19,20].

In addition to enabling the tracking of these conditions, smart technology can alert users and/or healthcare providers to health deterioration, which has the potential to lead to neurodegenerative health conditions like MS, or health conditions that require long-term rehabilitation, such as a stroke. People who experience these health conditions also often develop mental health issues, such as depression and anxiety, which can impair their quality of life [21,22]. Therefore, access to a smart device that enables the monitoring of stress levels or promotes wellbeing activities such as mindfulness may also be beneficial. The effectiveness of mindfulness for psychological symptoms and pain management has previously been shown for people with MS [21], so such components could be desirable features of smart devices aimed at holistically improving health and wellbeing.

### 1.2. Using Technology for Self-Management

In addition to the management of specific health conditions, people often use technology for self-management. The design of ASs for a plethora of different tasks enables users to carry out everyday tasks effectively and efficiently. This might include calendar and reminder apps, smart home technologies, financial, banking and shopping apps, and so on. Such technologies give more control to users and, consequently, lead to a reduction in stress. The use of ASs for home sensing and environment control, highlighted in Section 1.3, can also be incorporated into digital lifestyle management, giving people greater control over their home environments and creating better spaces for their health and wellbeing.

More directly related to the health context, ‘lifestyle management’, a form of self-management that involves implementing and/or maintaining lifestyle habits such as regular exercise, healthy diet, or reductions in stress, can help control symptoms and prevent certain health conditions [23]. It is a commonly used practice to help control and address symptoms of obesity [24], diabetes [25], hypertension [26], depression [27], PCOS [28,29], endometriosis [30], and other conditions. Lifestyle management can be used by itself or with other treatments, such as pharmacological and behavioural treatments, including intensive behavioural therapy (IBT) [23].

As behavioural approaches have limited access, can be expensive, and require users to engage face-to-face, the use of ASs for lifestyle management offers an alternative solution to the challenges involved in the sustainable and long-term implementation of lifestyle habits. These ASs tend to be based on goal setting and evaluation (via the quantified self), as well as on signals for encouragement and motivation (a challenge in the long-term adoption of habits) [31]. Traditionally, there are two main approaches to digital interventions for lifestyle management: app-based and web-based. Both approaches are adaptive and feedback- and multi-media-based; however, app-based approaches have the additional potential to integrate wearable technology, GPS data and chat boxes [31]. Other approaches include interactive TVs [32,33], voice assistants (VAs) [34], and smart mirror technologies [35,36].

This paper focuses on the latter, a smart mirror used to examine health monitoring and management. This differs from the wearable-type technology discussed so far in that it is embedded in the home. Smart mirror technologies fall into two camps: digital mirrors, which simulate mirrors on camera-enabled digital devices, such as tablets, phones and wall-mounted displays; and augmented mirrors, which enhance conventional mirrors with digital capabilities, such as by placing digital displays behind half-silvered mirrors, as explored here. Research into digital and augmented mirrors has explored a variety of applications, including motivating and guiding daily tasks. The AwareMirror [37] provides a personalised display during tooth brushing and the FitMirror aims to improve mood and fitness during morning routines [38]. Other uses include posture improvement [35], stress detection [36], and on-going medical monitoring [39]. With a reflective, mirror quality that enables the self-monitoring of physical abilities, an interactive interface, and additional health monitoring, along with the integration of additional data from other personal devices, such as smartphones and other wearables, for optimum decision-making, a smart mirror has the potential to effectively monitor health conditions in a novel way. Additionally, user wellbeing needs to be central in the mirror’s design, to promote psychologically respectful technological developments [40].

### 1.3. The Home as a Space for Wellbeing

The built environment strongly shapes social and individual behaviours and lifestyles, and it is a significant factor contributing to individuals’ health [41]. Recognising this important role, architecture and related building-design disciplines are moving towards the design of spaces for wellbeing. For instance, access to green areas and the integration of nature aspects within and surrounding buildings have been shown to improve and support physical and mental health [42,43,44]. The convergence of computing and architecture has created exciting opportunities for the creation of spaces where environmental conditions such as air quality, temperature and lighting can be sensed and adjusted to ensure comfort and ideal conditions for occupants [45,46]. Moreover, fast-paced advancements in sensing technologies embedded in the built environment, coupled with portable (e.g., mobile phones) or wearable (e.g., smartwatches) technology, have the potential to assist with the provision of healthcare. For example, there is a longstanding interest in embedding sensing technologies for monitoring people in care homes [47,48], especially when dealing with delicate and deteriorating conditions, such as dementia [49,50].

In addition, a shift is developing towards the design and adaptation of housing to support healthcare and wellbeing. The reasons for this are manifold: the ongoing strain in public health services [51,52], increasing populations in need of long-term support [53,54], the emergence of telemedicine and remote healthcare provision [55], and the increasing role of the home in modern lifestyles. The COVID-19 pandemic has increased the urgency of these adaptations; healthcare systems are still struggling to recover from the overflow of patients and lack of resources, and individuals continue to struggle with the aftermath of the pandemic and its effects on physical and mental health [56]. We still do not fully comprehend the consequences of the virus on the human body [57] and society at large. The numerous lockdowns and self-isolation periods also had damaging effects, both physically (e.g., decreased opportunities for moving and going out) and mentally (e.g., stress, anxiety and depression) [58,59]. Some argue that this pandemic has been a mass disabling event [60].

With increasing numbers of people studying and working remotely, attention must turn to how the home can promote wellbeing. Building design has moved towards the design of salutogenic living spaces, foregrounding wellness, comfort, fitness, and mindfulness. For instance, by maintaining air and water quality, promoting thermal and olfactory comfort (e.g., ventilation, easy dispose of waste), providing aesthetically pleasing and accessible pathways, stairs, fitness, religious or learning spaces, adjustable workstations, and so on. [61]. While buildings have been designed to passively increase or maintain wellbeing (e.g., open and flexible areas), digital technologies like smart mirrors and other smart devices in the home allow architectural spaces to make more active, potentially even proactive [62] contributions to health and wellbeing. Smart technologies embedded in the home can provide the opportunity to move beyond simple data collection for personal use towards providing active, real-time feedback and recommendations, or even interventions for occupants based on both general and individualised parameters. Nonetheless, the relationship between the home and the data collected needs to be carefully addressed, as in the contexts of other built environments that collect data from occupants [63,64].

## 2. Materials and Methods

At the core of this project is the consideration of the responsible ways in which technology should and could be developed to support healthcare at home, focused on maintaining the autonomy and dignity of people receiving healthcare. Therefore, principles of responsible research and innovation (RRI) [65] were embedded from the start. This was especially important given the multidisciplinary nature of the research team, who approached the project with different priorities and experiences [66]. One early exercise to establish common ground was the use of Moral-IT cards, which are designed to encourage reflection and engagement in ethics-by-design [67], to highlight potential ethical questions surrounding the development of technology. The team returned to questions posed by the cards often during the project, including prompts for discussion and points to consider when examining the results, as summarised in [66].

A principal component of RRI is the involvement of users from the beginning of the process. The team engaged with patient and public involvement (PPI) groups throughout each stage of the project to ensure that the focus groups reported in this paper were accessible and inclusive. Specifically, the input from the PPI activities was utilized to inform the study design, which accommodated the diverse needs of recruited participants with lived experiences of MS or stroke. The approach taken to PPI was in line with the UK Standards for Public Involvement in Research [68] while also being proportionate to the small scale of the project. Existing groups from each user population, who had links to the authors, were approached in an advisory capacity, and two online meetings were conducted lasting between half an hour and an hour. A member of the research team briefly outlined the aims of the project, the proposed methods for conducting the research, and the potential research questions. The remainder of the meetings were discussions, with group members providing feedback on the proposal and suggesting improvements. This helped the team to understand challenges these users had when engaging with digital technologies in general and during their care, which were subsequently incorporated into the study; for example, the MS group suggested that monitoring of walking, talking and gait or posture would be worth investigating with users, as they saw both positives and negatives. This was added to the discussion points in the focus groups’ plans and a section on posture was added to the scenarios. Both groups also pointed out practicalities for carrying out research, such as issues with online meetings, and the importance of safeguarding and debriefing if using potentially upsetting scenarios. This enabled the research team to design the focus groups in a responsible and appropriate manner. To address safeguarding issues, a member of the research team was present at each focus group specifically to monitor the chat function of the session, and participants were given specific instructions to follow if they were distressed at any point, detailed in Section 2.2. To address issues related to the meetings being held online, the facilitator also ensured that people with communication difficulties were able to contribute. Additionally, the durations of the sessions were increased to ensure there was ample time for rest breaks; one of the sessions was split into two as a result of these considerations.

### 2.1. Participants

Authors had existing links with a group of people with MS and with a group of people who had suffered a stroke. These groups were approached, and an online talk was given to raise interest in the project. The group members were then invited to declare whether they were interested in taking part. The majority of the PPI group and participants came from these groups. Others were recruited via social media and targeted emails to known groups and people. Participants were required to be over 18 years of age and either to have been officially diagnosed with MS or to have had a stroke at any point in the past. Participants provided consent by filling out an online form and were thanked for taking part with an Amazon voucher worth GBP 20.

#### 2.1.1. Group A: Management of MS

Six participants with MS took part in a single focus group, ranging in age from 38 to 63 (average age 53). They included 3 males and 3 females, and all participants were white; 4 participants had at least an undergraduate degree. Participants were diagnosed with MS between 3 and 14 years prior to the study (average 9 years); three had relapsing–remitting MS, one had primary progressive MS, one had secondary progressive MS, and one declined to state their condition. Two participants had additional conditions: trigeminal neuralgia and depression.

#### 2.1.2. Group B: Management of Post-Stroke Care

The stroke group took part in two one-hour focus group sessions, due to health considerations, and some participants took part in just one of the sessions. In total, 11 participants attended the first session, although 1 withdrew their consent later, and 13 participants attended the second session; 8 attended both sessions. They included 12 males and 3 females, and all were white. Eight participants provided additional information; ages ranged from 52 to 75 years (average age 58). Two participants had at least an undergraduate degree. All had experienced one stroke and time since stroke ranged from 1 to 16 years (average time around 6 years). Four participants stated that their etiology was an infarct, one stated that it was haemorrhage, and two stated that it was unknown. For 5 participants, the lesion was on the left side, for 2 it was on the right, and for 1 it was on both. Effects included a change in dominant hand, memory loss, problems with vision, communication problems, weakness, inattention, or paralysis on one side of the body, fatigue, limp or drop foot, muscle and neuropathic pain, and spasms.

### 2.2. Materials and Procedure

Sessions took place online through Microsoft Teams. All were attended by a facilitator who primarily led the sessions and asked the questions, and at least one other member of the research team who monitored the group and the meeting chat, and who took notes. Both groups were presented with the same materials and covered the same content; the only difference was that the stroke group took part over two sessions. At the start of the sessions, participants were assured that they did not have to discuss their medical history, or that of others, and that they were under no obligation to disclose any information they did not want to, that they did not have to provide any sensitive information, and that any identifying information would be removed. Participants were also free to leave and re-join the workshop at any time or to talk to one of the research team about any concerns; to ensure further safeguarding, if they were to type an ‘X’ into the meeting chat, a member of the workshop team would pick it up and the group would move on from the discussion immediately.

**Figure 1:**
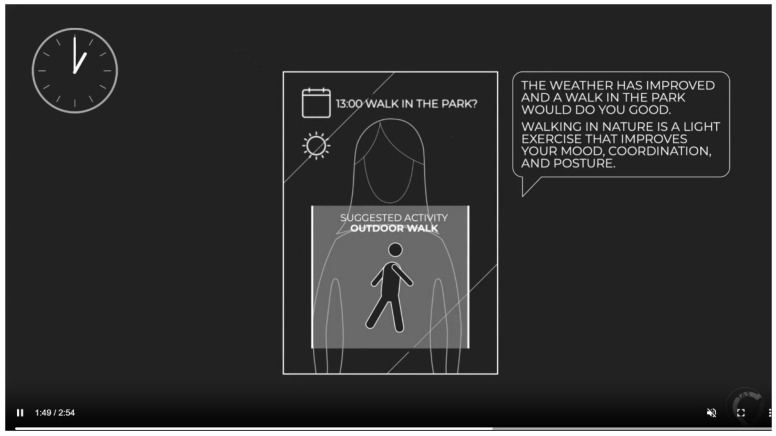
Screenshot from the positive-scenario video.

The first part of the session started with an introduction to ASs and how they may be used for health, as well as an overview of the project. This was followed by an open discussion about participants’ current use of technology to support their health and wellbeing. The second part of the session focused specifically on smart mirrors, with an introduction to smart mirrors, including a video illustrating an existing example. After a brief discussion about the potential use of this technology, two scenarios were presented in turn. This method was drawn from approaches such as the ‘ContraVision’ framework by Mancini et al. [69], which present both positive and negative narratives to participants in order to provoke a wider spectrum of reactions than is possible with a sole scenario. Participants were shown a ‘positive scenario’ video describing a day-in-the-life using the mirror for either stroke rehabilitation or monitoring of MS, in which everything that took place was framed in a positive way. The transcript for this video is provided in Appendix A and illustrated in Figure 1. Participants were asked to discuss their feelings about the mirror based on this video. The transcript is as follows:

*“Jenny has a smart mirror installed in her home. She uses it for monitoring her health after she had a stroke. Every morning, when Jenny wakes up, she activates the mirror, and it greets her with a friendly ‘hello’ and a summary of the day ahead for her. Throughout the day, the mirror helps out by providing reminders for events by linking to Jenny’s calendar, recognising when Jenny is standing in front of it and monitoring posture and facial expression, making suggestions for what to do based on the data it has collected over time, and guiding Jenny through daily exercises and mindfulness activities. At 10 am, the mirror reminds Jenny that it is time for her to take her medication. It also reminds her to order another prescription and offers to do that for her. Later in the day, when Jenny enters the bathroom, the mirror praises Jenny for the improvement in her posture and asks how she is feeling today. After lunch, the mirror suggests a nice walk as the weather is good and the data shows that a few short walks a week is good for Jenny’s symptoms. At 4 pm, the mirror suggests some gentle exercises to maintain her good posture. It guides Jenny through them a couple of times to make her comfortable. Then it does a short meditation with her to wind down. At the end of the day, the mirror prompts Jenny that it is close to her bedtime and suggests some calming activities to wind down. Before bed, the mirror displays the day’s data to Jenny and asks her if she would like to share it with any of her designated contacts”.*

**Figure 2:**
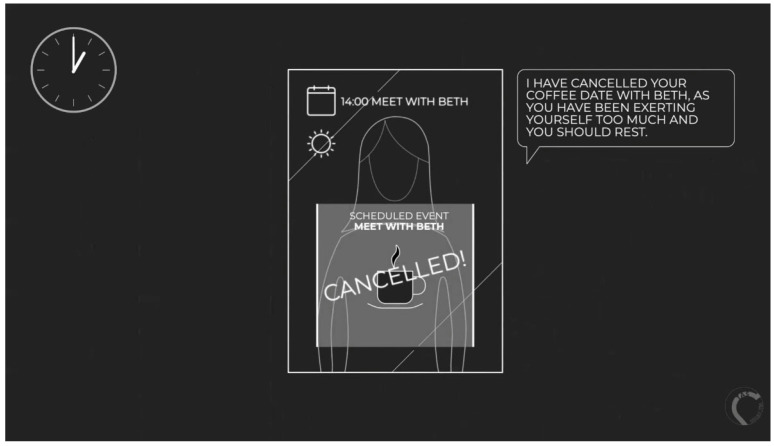
Screenshot from the negative-scenario video.

The participants were then presented with a ‘negative scenario’ video describing the same activities, but with a negative framing (Appendix A, Figure 2). Participants were again asked to discuss their feelings about the mirror. The transcript is as follows:

*“Jenny has a smart mirror installed in her home. She uses it for monitoring her health after she had a stroke. Every morning, when Jenny wakes up, the mirror recognises that she is up and promptly provides a list of things to do for the day ahead of her. Throughout the day, the mirror provides regular notifications which Jenny must respond to. It provides reminders for events by linking to Jenny’s calendar, recognises when Jenny is standing in front of it and monitoring posture and facial expression, makes suggestions for what to do based on the data it has collected over time, and guides Jenny through daily exercises and mindfulness activities. At 10 am, the mirror tells Jenny that it is time to take her medication. As she does not immediately respond it prompts her several more times. Later in the day, when Jenny enters the bathroom, the mirror berates her and tells her that her posture has gotten worse. It tells her that she should check in more often. After lunch, the mirror tells Jenny to cancel her plans as she has been overdoing it. It removes a coffee date from her calendar. At 4 pm, the mirror tells Jenny that she should do some extra exercises due to her posture measurements earlier. It skips the mindfulness activities for that day, prioritising the exercise. This cannot be overridden. At the end of the day, the mirror tells Jenny that it is her bedtime and begins a countdown for turning off the television and main lights. Before bed, the mirror sends all of the data it has collected over the day to the manufacturer of the mirror and to Jenny’s GP”.*

The session ended with a general discussion of AS for healthcare and a summary of the benefits and drawbacks of the smart mirror.

### 2.3. Analysis

Focus groups were recorded in Microsoft Teams. The audio was extracted and transcribed using an automated transcription service, a service provided by the institution of the first authors for use by researchers. Therefore, it is GDPR-compliant and participant data were protected. The transcripts were not perfect, however, so they were checked and corrected by three researchers, who thoroughly listened to all recordings and ensured accuracy. Next, the data were thematically analysed. The analysis was completed on Microsoft Excel and followed the process defined by Braun and Clarke [70] consisting of (1) familiarisation with the data, (2) initial code generation, (3) theme search, (4) theme review, (5) naming and definition of themes, and (6) report write-up. Separate initial analyses for each user group were carried out to avoid conflation of health conditions and missing relevant information particular to each group. Each separate analysis was then merged to generate one single set of themes covering both workshops. That is, steps 1–3 were conducted separately for each group, and once initial themes were developed, initial groupings were reviewed and combined to produce the final themes presented in this document. Throughout the paper, participants are referred to with a number (P1, 2, 3…) and with S if they were in the stroke workshop (P1S) or M if they were in the MS workshop (P1M).

## 3. Results

**Figure 3:**
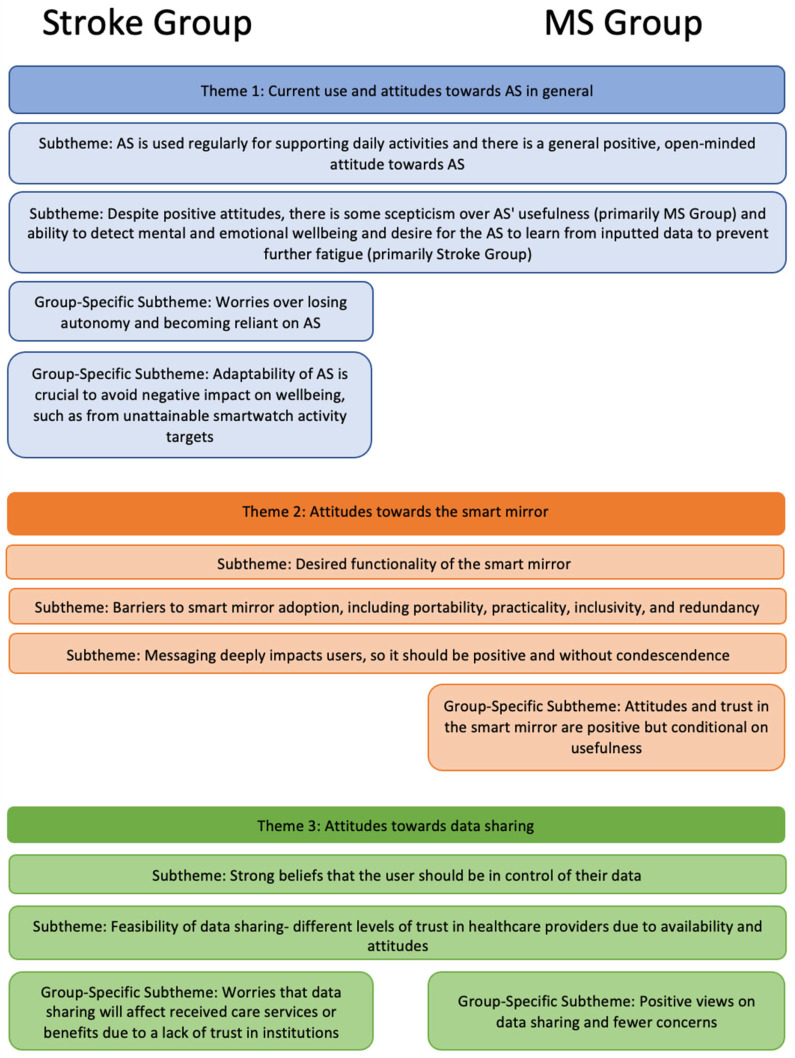
Summary of the themes and subthemes developed from the focus groups. Subthemes that spread across both groups horizontally refer to those found in both participant groups and subthemes placed under one group only represent group-specific subthemes.

Three major themes and eleven sub-themes were developed, as summarised in Figure 3 and described below. Note that some subthemes were only identified within a specific user group, as indicated in the figures and their descriptions.

### 3.1. Theme 1: Current Uses of and Attitudes towards ASs in General

The first theme refers to the use of ASs in the participants’ day-to-day lives and their overall feelings towards them. The participants reported regularly using a range of ASs for a variety of purposes, and they expressed interest in and curiosity towards future ASs that could help support their health conditions (Figure 3: subtheme 1a). Several participants appreciated the value of devices such as smartphones and smart speakers for setting alarms and reminders for daily tasks, such as taking medication, and for using calendars and notes for planning activities; these all helped with the memory problems experienced by many in both groups.

*“And then the other aspect of my MS is the fatigue, so I do have a smart watch which I wear to track how much I’m walking and how active I am, but also to track my sleep… so sometimes when you go through a week you know you there will be days where you feel really wiped out and it’s good just to be able to check, you know, have you been walking too much? Have you not had enough sleep? But also to, you know, if you’re going out of pattern, [it’s] probably worth having a conversation with your GP or a specialist”*

[P3M].

Apps for brain training and meditation were also used by a couple of participants to support their cognitive and mental wellbeing. Although there was a low uptake of these apps among the rest of the participants, there was general agreement that they sounded useful and effective. The participants who used smartwatches or fitness wristbands to monitor their daily activities, such as walking and sleep, expressed the usefulness of observing their own patterns; although the AS does not give personalised feedback (e.g., detecting or preventing unusual levels of activity), the participants recognised their own thresholds and patterns in the data collected by the AS and used them to better understand, for example, their fatigue. They also reported the adoption of smart home technology for monitoring and controlling their environments, such as lightbulbs, plugs, thermostats, and doorbell cameras. These technologies are often used in multi-person households, so although they are not necessarily intended for the monitoring users’ health, some of the participants felt that they provided autonomy and support, especially to those with limited mobility.

*“It doesn’t monitor my health at all, it’s just to make my life easier. In controlling the aspects of my home, I’m completely chair-bound, wheelchair-bound. I can’t walk at all. And I’ve really only got the use of one arm, so anything I can use which helps me control things is really useful. I’m on my own occasionally. My wife’s my carer. She’s around most of the time, but she has to go out every now and again. Um, so for when she’s out, it’s the only way I’ve got of controlling my home”*

[P1M].

When the participants recognised shortcomings in the systems that they regularly used or gaps not yet addressed by technologies, they had generally positive attitudes towards future ASs, suggesting ideas related to the automated detection of personal patterns, to notify users when sleep or activity exceeds certain thresholds, help to prevent fatigue, and further support their care and carers.

However, some people were skeptical about the capabilities of current and future ASs (Figure 3: subtheme 1b). Some of the participants entirely dismissed certain ASs as inappropriate for supporting their health. For example, smartwatches and fitness wristbands were seen as pointless for those whose mobility was severely reduced. Some people in the MS group declared they did not know of any technology that could support their MS symptoms, despite being generally open-minded towards ASs and keeping themselves up to date with recent advancements. There was particular skepticism about detecting and supporting non-physical states. A salient topic throughout the sessions was the fatigue experienced by the participants. For example, the participants in the stroke group extensively discussed ‘neuro-fatigue’ as commonly resulting from experiencing a stroke. There was a consensus that it would be highly valuable to have ASs that could help to prevent and ease physical and mental fatigue, as well as emotional distress.

*“I think this is where it gets really complicated because it’s not just high activity or what you do in a day. So, I consider myself quite able physically and communication-wise, and I feel very fortunate for that but you saw me really struggle with the word ‘alarm’ today. Now that is because I’m really tired at the moment. I’ve had a lot going on in my head and physically for various different reasons”*

[P6S].

However, the participants also found it difficult to imagine how fatigue symptoms or emotional conditions like depression could be detected by the AS. Participants in both groups suggested that future ASs should allow users to manually enter data about their emotional and mental states, so that the ASs can learn from these data and subsequently help to monitor and support their users. The participants further proposed systems that could prompt they entry of data, like a diary, using metaphors such as ‘battery level’ to explain their energy states.

*“So, a regular person, a non-stroke survivor, starts with a fairly full battery at the beginning of the day, whereas a stroke survivor typically starts with less* […] *Because of that, the stroke survivor, the levels of fatigue kick in sooner than someone else’s. And then you’ve got the added complication of neuro-fatigue. But if there was a way to potentially have that information, that whether there is qualitative or quantitative data information in the system somehow, so the system then learnt by that, urm, and then set you goals which took that into account, that would be, I mean, it would be really clever”*

[P1S].

Additionally, there were two other subthemes specific to the stroke group. In contrast to the people in the MS group, who saw their technology use as a way to gain independence and support, the participants in the stroke group expressed strong concerns about losing their autonomy to ASs if they depend too much on them (Figure 3: subtheme 1c): “*It’s more important for you to be independent than use this device obviously*” [P5S]. Although they felt that technology is useful, it was important for them to complete tasks for themselves and push themselves physically and mentally to avoid becoming reliant on technology.

*“You mustn’t have these, too many of these benefits and these machines, otherwise you’ll be sitting in the chair all day and it’ll make a cup of tea for you”*

[P3S].

This could be explained by the physical differences between the people in each groups, since those in the MS session reported more constrained mobility than those in the stroke group. Additionally, the stroke participants often talked about having to re-learn tasks and re-wire their brains, which they felt would be more challenging if they relied on technology for common tasks.

Lastly, the participants in the stroke group profoundly remarked on the need to integrate adaptability into AS design (Figure 3: subtheme 1d). Whilst the smartwatch users reported that tracking their daily steps could evoke feelings of happiness and pride, the participants in the stroke group pointed out that the pre-set goals could seem unattainable and exert negative effects on their emotional wellbeing.

“*You’re going to think ‘well I should be aiming for this’, and ‘I can’t even do this’, so I think achievability* [is key]*, and that’s going to be individual obviously to the person because it’s depending on everybody’s needs”*

[P6S].

These experiences became annoying and frustrating for the participants and threatened to push some to give up using the devices altogether. Adaptability could be based on the system learning automatically, but the ability to manually change goals and ‘correct’ the assumptions made by the AS was highly important. The design of this flexibility needs to involve not only the differences between individuals, but also how their physical and mental conditions change from day to day.

*“I think also [name], you know, what you’re able to do on Monday may drastically vary on another day, mightn’t it. So, if you’re not able to change the goals and it’s changing them for you, one day I’m capable of doing loads and another day I’m not. I’m done by about 11 o’clock”*

[P5S].

### 3.2. Theme 2: Attitudes towards the Smart Mirror

The second theme encompassed the participants’ feelings and attitudes towards the concept of the smart mirror. A range of desired functions was expressed (subtheme 2a). Both participant groups believed that the smart mirror could potentially be especially useful in identifying and monitoring memory problems (“*You know it’s like… it’s handy for those that’ve got a memory problem*” [P8S]) and mobility issues (“*As a prompt, it would be really good… I go far too long with a warped body, as it were*” [P2S]). The participants had a range of personal stories that suggested the usefulness of mobility monitoring. One participant, who reported spending long periods of time without seeing anyone else, suggested that the smart mirror might diagnose mobility issues affecting posture or gait that would otherwise be missed until the next appointment with a healthcare provider.

Another desired smart-mirror feature was the combination and summary of information from multiple sources and devices, including data pulled from external sources, such as NHS patient records, individuals’ other devices, and self-reported input.

*“I think that numerous data sets plus some way of getting in your NHS diagnosis—where you are on the scale for your different attributes. If all of those datasets feed through, I can see it actually being really, really helpful”*

[P3M].

This would also save the user from having to check multiple devices throughout the day for alarms, reminders, and others. The participants also highlighted the multiple input methods that were needed by those with a variety of physical problems, such as voice-activation for those experiencing tremors, poor dexterity, or other physical disabilities, and alternative activation functions for those experiencing speech difficulties. While most of the participants had largely positive views on the smart-mirror concept and proposed ideas about what would make it useful, there were inevitably views expressing concerns about issues that could hinder its adoption (Figure 3: subtheme 2b). For many of the participants, the mirror’s fixed location would be a major issue affecting its usefulness, as the AS would fail to capture activity in spaces away from the smart mirror, making it easy to mislead the smart mirror by behaving differently when in front of it. To circumnavigate these issues, multiple smart mirrors would be needed around the home, which may be expensive and impractical. The participants suggested that it would be crucial to have the smart mirror linked to other, more portable devices. Many of the participants expressed concern over the practicality of setting up the smart mirror if the user were elderly or had a disability, as not everyone would have someone to help them. Moreover, sufficient space would be needed in the home to complete exercises in front of the smart mirror, making it less inclusive for those in small dwellings.

*“I think it’s really important* [name] *saying how he’s got a small space, so if it was to be a mirror, it wouldn’t really work for him. And this is where my concern would be, that it’s not all inclusive, and I think it’s a really, really great idea, but I think the mirror idea needs possibly to be scrapped because I don’t think that makes it accessible for everybody”*

[P2M].

There were also concerns that the mirror could not provide value to users at all the stages in their respective medical condition journeys, regardless of whether they had regular check-ins with care providers, or for those with different health needs. The final drawback discussed by both groups was the issue of technology redundancy and the feeling of being overwhelmed by multiple devices. Multiple participants felt that the smart mirror was not distinguishable in function from other ASs, such as smartphones, smart speakers like Alexa, or tablets.

*“I know my health flags; I know I’ve got my computer. I’ve got an Alexa that does most of that and a little screen on it. So, I don’t understand what purpose there is* [to the smart mirror]”

[P8S].

Additionally, the participants expressed that having multiple technological devices along with the smart mirror would be overwhelming and add to the mental fatigue felt by individuals with a medical condition, such as MS, by or those recovering from a stroke. The participants felt that in any case, technology is taxing for older generations and that, with the addition of a medical condition that involves fatigue, the idea of using a smart mirror along with other devices seemed very overwhelming and unnecessary.

In addition, the participants emphasised that messaging should be framed to avoid condescension or disrespect (Figure 3: subtheme 2c). For those with a medical condition or who have experienced a life-changing medical episode, there are consequences that can seriously affect mental wellbeing, including lower confidence and a propensity towards depression and low mood. Therefore, the tone and content of messaging from the smart mirror should be positive, gentle and encouraging to benefit wellbeing, rather than an instructing, condescending, or demanding tone, which may have a negative impact on users’ mental health and wellbeing.

*“It would reduce me to tears if it didn’t have a very gentle approach, because when I’m having my bad days I already know I’m having a bad day and I know I can’t get a coffee with my friend and that’s horrible as it is. I don’t need some flipping gadgets saying “and don’t do this”…I know that I’d be really upset and it would probably put me to the floor to be honest with you”*

[P2M].

*“I think there’s also something about how the, what the content of them—the message, whether it’s a text message, whether it comes up on your phone. So, if you were expecting to hit 5000 steps because you have, you had like normal days before and you didn’t quite do it, it should say in that sort of way, it should have a positive spin on it”*

[P1S].

Finally, specifically in the MS group, the participants felt that attitudes towards the smart mirror would depend largely on its usefulness to the individual (Figure 3: subtheme 2d). For example, some participants would want a trial period before purchasing the smart mirror to assess its usefulness before committing to the technology. Similarly, the participants’ trust in the smart mirror would be conditional on their trust in source of the technology and the data it used. For example strong trust would be placed in data from the NHS.

*“Well, I would assume that it’s coming from a trustworthy source, whoever’s programming it, is that coming from the NHS? Or who’s putting information into it?”*

[P2M].

### 3.3. Theme 3: Attitudes towards Data Sharing

The third theme combined the different views on data sharing within the context of health monitoring. Overall, there was a willingness to share data with healthcare professionals if they were under the control of the participants (Figure 3: subtheme 3a), as different people felt comfortable sharing different types of data under diverse circumstances.

*“I think it has to be the user. That’s what personally I would think because somebody else could abuse the situation”*

[P7S].

The conditions that would have to be met for different participants to be willing to share their data ranged from the ability to review the data before sharing them with healthcare professionals, the option to opt out of sending data to GPs, the anonymization of data before sharing, and the option to only share specific types of data.

*“You need to have some control over it, either to be able to say ‘no, I don’t want the data to go’… or to know that it’s gonna go by the right routes and be anonymised”*

[P4M].

Some of the participants only felt comfortable sharing their data with specific healthcare professionals (for example, with physiotherapists, but not with GPs). While the specific conditions varied, all involved giving control of the data to the user.

Participants from both groups also raised points regarding the feasibility of data sharing (Figure 3: subtheme 3b). There were concerns that healthcare professionals would not have time to review any of the data that were shared.

*“But how often is it, the doctors are very busy, would they have time to look at it? If they’ve got ten people on their books that have got a mirror, and they’re all supplying data, how do they deal with it? They’re overstretched as it is”*

[P8S].

For the participants, if data were to be shared with healthcare professionals, there was an expectation that these data would be used to inform their individual treatments.

*“I want the NHS to actually be doing something on the other end rather than just providing data to them for them to collate. I want it to go to, you know, I want it to go to my consultant. I want it to be looked at for my annual reviews and things like that. I’d like it to go to the GP because you know, if it’s not being used for beneficial purpose, there’s really not much point giving it to them”*

[P3M].

The variation in the attitudes towards data sharing is further seen in themes 3c and 3d. Some of the participants from the stroke group had specific worries about the consequences of data sharing with medical institutions (Figure 3: subtheme 3c). They expressed the concern that if the data that were reported were interpreted as suggesting that the patients did not need health or economic support, they may not receive access to services such as physiotherapy appointments or government benefits.

*“I think you’d have to be very careful though because it might catch you on a real good day, you know, and sort of take that as an example, report it back to your doctor. Then all your benefits go, if you’re on benefits of any sort, you know”*

[P5S].

A lack of trust was also conveyed as the participants questioned the intentions of the mirror manufacturer and the need for manufacturer to collect personal data, which had a subsequent effect on the willingness of participants to share their data. Interestingly, these concerns were only mentioned by participants from the stroke group, whereas the participants in the MS group were more open-minded towards data sharing (Figure 3: subtheme 3d), although this was once again conditional on the usefulness of the data to the individuals and whether they benefitted others and the medical community. A few participants had taken part in medical research for several years; thus, they felt comfortable sharing their data in the hope that it could advance scientific knowledge and improve support for people with their condition in the future. Moreover, they reported being happy to share their data with their healthcare professionals to improve their own care. However, they did not approve of having their information shared with other stakeholders or of its being sold to third-party companies, especially if they were not anonymised.

*“I was gonna say for me personally I have no problems with the NHS having my full data, it’s when it gets outside of the realms of the NHS, I’m not comfortable with the data being shared. It would have to be anonymous outside of the NHS”*

[P3M].

## 4. Discussion

Using design fictions, our study, involving people with MS and those who were recovering from a stroke, inquired as to the participants’ current use of technology in their healthcare regimes and their attitudes towards the future integration of autonomous healthcare systems, especially smart mirror, into the home. Our findings describe a complex landscape of advantages and disadvantages in tension with each other, the intricacies and details of which we discuss and synthesise into design recommendations below.

### 4.1. The Current Use and Perceptions of AS

Many of the participants already made use of a range of technologies to control and monitor their homes, such as lightbulbs, plugs, thermostats, doorbell cameras, and other smart home technologies. These devices helped them to feel a sense of autonomy and support, especially those who had limited mobility or other physical issues. This is consistent with findings reported by Ayobi et al. [17], showing that people with health issues can benefit from ASs and self-tracking behaviours due to the sense of control they gain over unpredictable conditions. However, technology was not a solution for all the participants, as some of the MS participants found ASs to be inadequate to their circumstances. This showcases the importance of the user-centred design of ASs for healthcare, integrating the specific circumstances of users and the wider context, enabling and enhancing their personal health experiences [71]. An important point that technology designers should consider is that the overuse of and overdependence on smart technologies may lead to a loss of autonomy and become detrimental to health. Hence, users may be reluctant to include any additional technologies in their routines.

#### The Ecosystem of Devices Used for Lifestyle and Self-Management

Alarms and reminders for daily tasks, such as remembering to take medication, as well as calendars and notes for planning activities, helped many of the participants to deal with memory problems. Brain training and meditation to support cognitive and mental wellbeing, whilst having low use, was received positively, resonating with the desire for mindfulness activities in health apps discussed by Di Cara et al. [21]. The monitoring of daily activities, such as walking and sleeping, was also common. However, in most cases, these technologies were not considered in terms of ‘healthcare,’ with the participants often stating that they did not use any technology to support their physical or mental health.

Combined with the range of in-home devices in use, it is clear that the participants created an ecosystem of devices that helped them to manage their day-to-day lives and their health conditions. On the surface, this seems to suggest a form of ubiquitous computing similar to Mark Weiser’s vision [72], with a multitude of (networked) computing devices within reach at all times. However, unlike in Weiser’s vision, there was a clear distinction for the participants between lifestyle-management technologies and specific healthcare devices. Some, especially in the MS group, stated that they did not know of any technology that could alleviate their symptoms, despite using a range of devices and having a generally open mind towards future developments. This highlights how the quantified self is not currently accessible to all people, potentially rendering some “statistically invisible”, depending on how AS data are used. It also highlights an opportunity for individuals to form clearer links between lifestyle management/their quantified selves and their healthcare regimes by (1) increasing applications to different symptoms, (2) more directly connecting and contributing to individuals’ healthcare, and (3) connecting different devices to increase their value in users’ healthcare regimes.

### 4.2. The Addition of Smart Mirrors to Support Physical and Mental Health

A major point that emerged was that each patient has a unique journey through their health diagnosis, and individual experiences during each stage of a medical condition need to be considered in the design of ASs. These ASs must be flexible and inclusive to adapt to users’ unique and changing abilities, which are associated with the presentation of their condition and its management. While the smart-mirror technology is adaptative [31], it is currently only used within a very constrained setting. The attitudes towards the smart mirror depended greatly on its perceived usefulness in the specific healthcare journey of each individual. Overall, technologies such as smart mirrors have the potential to help with daily life and reduce stress and neuro-fatigue, in turn improving general wellbeing and having positive effects on medical conditions such as MS. However, the design of these technologies must consider the specific use cases of those who can benefit from them the most, and how these benefits can be achieved in a way that is non-fatiguing. This could be achieved by technology being unobtrusive and operating in the “background”, which would “not require active attention”, as described by Weiser [72]. Moreover, any health technology should take into consideration external economical, physical and social factors [73].

Whilst both groups felt that a smart mirror might potentially be useful to those with memory problems, and as a form of lifestyle management to prevent and support fatigue, the major appeal of the smart-mirror concept was in physical therapy, gait analysis and other forms of physical support, especially between healthcare appointments. This is echoed in the literature, given that the development of ASs for the conditions studied so far focus on mobility and balance, gait and movement analysis, and limb rehabilitation [16,18,19,20]. Given the desire to avoid excessive reliance on technology, such features should be designed to be non-addictive, with the aim of training users to not need the technology.

The relationship between technology, health professionals, patients and wider health considerations is also highly important. Technology like smart mirrors may not provide value for users who do not have regular contact with care providers, including GPs and physiotherapists, who could oversee the use of these mirror and input or modify care plans. Even for those who do regularly see care professionals, the technology is seen as inconsequential if it is not used by these care providers to improve their individual care plans. There was an overwhelming sense that health institutions do not have the capacity to undertake this approach to health, since they are excessively overstretched to have time to review data; it is therefore important that ASs are designed to support and not overwhelm healthcare professionals, as well as users. This can be achieved through automated visualisations, ASs that can highlight changes and anomalies, and that can connect to health records and reports on patients to provide useful overviews/dashboards. The implementation of ASs should also be performed with a consideration of patients’ wider systems of care delivery and resources. The participants were worried that any data sharing could be used to remove rather than support their care, such as by making them lower priorities for appointments, or to deny their claims for government benefits. These last two points illustrate a lack of trust in government and (healthcare) institutions, rather than the technology itself, which must also be addressed.

#### 4.2.1. Recommendations for Necessary Technical Features of a Smart Mirror

**Figure 4:**
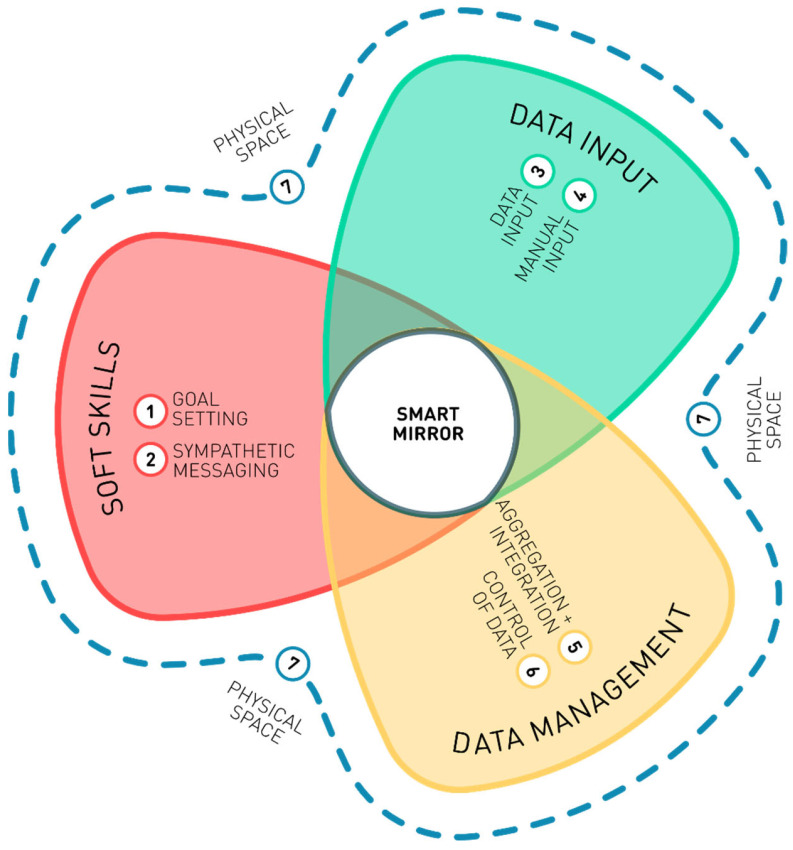
Design recommendations. A smart-mirror design needs to consider “soft skills”, “data input”, and “data management”, as well as the placement of the smart mirror in “physical space”.

The participants made a series of recommendations for features that would be necessary for smart-mirror adoption, as shown in Figure 4.

1. Goal setting. The goals of activities and other tasks should be user-led and variable based on user data, as autonomous goal setting is often tailored to the average individual and does not consider symptoms such as fatigue, which can lead to a negative impact on wellbeing for the user if the goal is unattainable.
2. Sympathetic messaging. Messaging should be framed as sympathetic, uncondescending/respectful and encouraging. The participants emphasised that people with health concerns often have lower confidence and may be more prone to depression and low mood and, therefore, that instructional or demanding messaging may have negative effects on users’ mental health and wellbeing.
3. Input methods. It is vital that smart mirrors support a variety of input methods, such as voice activation, keyboards and touch screens, to support people with speech problems, tremors, poor dexterity and so on.
4. Manual entry. Smart mirrors should allow the manual entry of emotional and mental states, including fatigue, with prompts to enter data, such as wellness levels at the start of the day and at subsequent points throughout the day. These data need to be aggregated with automatically collected data so that ASs can learn about the users’ needs. This also highlights the need for technology to be user-led and for it to enhance rather than undermine autonomy.
5. Aggregation and integration. A smart mirror should connect to, aggregate and display data from other sources, including other devices, patient records and manual inputs, to automate the detection of individual patterns and notify users when patterns change or when thresholds are reached. Such seamless integration could help to prevent fatigue and support care management by saving the user from having to check multiple devices throughout the day for alarms, reminders and other features.
6. Control of data. The sharing of data should always be under the control of the user, including which data to share and with whom, as well as levels of anonymisation and aggregation.
7. Physical space. A smart mirror should be physically and functionally accessible. It should be located in a space that is appropriate for it to function. For example, for the assessment of gait or posture, there should be sufficient space in front of the mirror for a person to walk or stand at the required distance.

#### 4.2.2. Perceived Barriers to Use of Smart Mirrors

The participants also highlighted several potentially adverse or unexpected outcomes, which could be barriers to the use of smart mirrors.

1. Lack of portability and practicality. The mirror’s fixed location is a major issue in its perceived usefulness, with concerns that it would fail to capture important activities. Multiple smart mirrors would be needed around the home, which may be impractical and expensive; hence, there is a need for smart mirrors to be linked to other, more portable devices.
2. Lack of accessibility and inclusivity. Setting up a smart mirror may be difficult or impossible for users who are elderly or disabled in any way, as not everyone has someone to help them set up a smart mirror. In addition, smart mirrors also require a sufficient amount of space to collect certain data, limiting their inclusivity.
3. Redundancy and the overwhelming nature of multiple devices. There was a strong sense that smart mirrors would be redundant, given that most people already have multiple technological devices that perform the same functions, and that the addition of more devices would be overwhelming and add to mental fatigue throughout the day.
4. Lack of trust in data sharing. The participants had strong feelings about sharing data with healthcare systems, including a fear that such sharing may lead to the removal of vital services. This lack of trust was also evident in sharing with others, outside of the healthcare setting, especially device manufacturers.

## 5. Limitations

There were a few limitations to the way in which the study was carried out. Due to the pandemic, the focus groups had to be carried out online, which may have excluded some participants, and restricted the number of those who could take part. For example, in both the PPI groups and the focus groups, people who were profoundly aphasic were not able to take part. This may have had an impact on the data collected, potentially missing important insights into the use of technologies in the home that could aid people who experience more severe health challenges. Furthermore, some of the participants had communication difficulties in the sessions. These people were given extra time to respond to questions and, where appropriate, other members of the group assisted in ensuring that the voices of these people with communication difficulties were heard; their responses were summarised and reflected back to them to check their agreement. This inclusive approach was used to try and ensure optimal participation by all the contributors; however, detail and clarity may have been lost due to impaired communication. Additionally, whilst the method of using a positive and negative scenario is well founded, the high levels of negativity in the second scenario could have elicited excessively unfavourable responses. However, the inclusion of a positive scenario may also have prompted highly favourable responses, so it is hoped that the overall response was balanced and accurate. The participants responded to parts of each scenario that stood out to them based on their own experiences. Further work to implement this ContraVision approach in the design and evaluation of technologies for health and wellbeing could test the efficacy of this novel method. Finally, although additional steps were taken to enhance the quality of the analysis, such as researcher reflexivity and the production of an electronic trail, the thematic analysis technique has limitations. These include the limited transferability of the findings (due to the small sample size of the MS group in particular) and the possibility of the researchers’ personal views and experiences subtly influencing or biasing the data analysis.

## 6. Conclusions

New appliances, such as smart mirrors, offer opportunities to support healthcare at home. However, as our stakeholder engagement revealed, this technology requires careful design in terms of hardware (e.g., the device’s precise location in the home) and software (e.g., responding appropriately to highly personal and variable health conditions). Our study was limited to two patient groups of people with MS and people who had experienced a stroke. Engagement patient groups with different conditions and focusing on a diversity of AS systems is likely to extend and help to make our design recommendations more comprehensive and robust. The next step in our research will be the design and physical deployment of a smart mirror in a real-world context to test our design recommendations.

## Data Availability

Qualitative data (transcripts) from the focus groups are unavailable due to privacy and ethical concerns.

## Appendix A

### Appendix A.1. Positive Scenario Video

The video presentation of this scenario can be seen at https://tas.ac.uk/wp-content/uploads/2022/01/Stroke-positive.mp4 (accessed on 16 August 2023).

### Appendix A.2. Negative Scenario Video

The video presentation of this scenario can be seen at https://tas.ac.uk/wp-content/uploads/2022/01/Stroke-negative.mp4 (accessed on 16 August 2023).
